# Using Brainwave Patterns Recorded from Plant Pathology Experts to Increase the Reliability of AI-Based Plant Disease Recognition System

**DOI:** 10.3390/s23094272

**Published:** 2023-04-25

**Authors:** Yonatan Meir, Jayme Garcia Arnal Barbedo, Omri Keren, Cláudia Vieira Godoy, Nofar Amedi, Yaar Shalom, Amir B. Geva

**Affiliations:** 1 InnerEye Ltd., Herzliya 4676670, Israel; 2 Embrapa Digital Agriculture, Campinas 13083-886, Brazil; 3 Embrapa Soybeans, Londrina 86085-981, Brazil; 4 Department of Electrical and Computer Engineering, Ben Gurion University, Beer Sheva 8400101, Israel

**Keywords:** electroencephalogram, digital images, labeling, soybeans, active learning

## Abstract

One of the most challenging problems associated with the development of accurate and reliable application of computer vision and artificial intelligence in agriculture is that, not only are massive amounts of training data usually required, but also, in most cases, the images have to be properly labeled before models can be trained. Such a labeling process tends to be time consuming, tiresome, and expensive, often making the creation of large labeled datasets impractical. This problem is largely associated with the many steps involved in the labeling process, requiring the human expert rater to perform different cognitive and motor tasks in order to correctly label each image, thus diverting brain resources that should be focused on pattern recognition itself. One possible way to tackle this challenge is by exploring the phenomena in which highly trained experts can almost reflexively recognize and accurately classify objects of interest in a fraction of a second. As techniques for recording and decoding brain activity have evolved, it has become possible to directly tap into this ability and to accurately assess the expert’s level of confidence and attention during the process. As a result, the labeling time can be reduced dramatically while effectively incorporating the expert’s knowledge into artificial intelligence models. This study investigates how the use of electroencephalograms from plant pathology experts can improve the accuracy and robustness of image-based artificial intelligence models dedicated to plant disease recognition. Experiments have demonstrated the viability of the approach, with accuracies improving from 96% with the baseline model to 99% using brain generated labels and active learning approach.

## 1. Introduction

According to the Food and Agriculture Organization (FAO), plant diseases cause more than 14% of crop losses worldwide [1]. Detecting and monitoring crop diseases are arguably the most important activities in agricultural settings around the world. Scouting is still carried out visually for the majority of cases. However, timely coverage of vast areas is often unfeasible, especially considering workforce shortages observed in many areas [2]. In addition, laboratory analyses such as molecular, immunological, or pathogen culturing-based approaches are often time consuming, failing to provide answers in a timely manner [3].

In this context, it is no surprise that significant research efforts have been devoted to the development of methods to automate the process [4]. Artificial intelligence techniques have been used to explore the information contained in digital images for many years, but the rise of deep learning models in the first half of the 2010 decade has caused an explosion in the publication of articles using this type of approach [5,6,7,8,9,10,11,12,13,14,15,16,17,18,19,20]. Although the number of articles continues to grow, progress on the subject has been slow. There are a number of reasons for this, including the adoption of similar approaches and the use of datasets that cover only a very small fraction of the variability associated with the problem [4]. As a result, methods and techniques dedicated to plant pathology found in the literature can rarely be applied under real conditions.

Building comprehensive datasets for plant disease applications is particularly challenging because it is not only difficult to collect images in the field, but there are several factors that introduce additional variation, including background characteristics, illumination conditions, geographic differences, and cultivar, sensor, and camera configuration, among others [21]. As data sharing becomes more common in the scientific community, the problem will tend to decrease, but significant gaps in the data representativeness will likely continue to exist for a long time. Techniques such as domain adaptation [22] and zero-shot learning [23] have shown some promise to increase the robustness of trained models to new data, but these are context dependent and do not work if the characteristics of the new images depart too much from those used to train the models [22]. Thus, the objective of creating models truly applicable to real plant pathology problems remains largely elusive.

With imaging devices becoming ubiquitous, it would be reasonable to assume that, with proper coordination between researchers, farmers, and field workers, it should be possible to overcome this problem. However, supervised learning requires that data be properly labeled before being used for training artificial intelligence (AI) models. This means that not only it is necessary to capture the images, but also that these images usually need to be analyzed individually by someone knowledgeable enough to reliably assign labels to each image. This process is labor- and time-intensive, which can become a significant hurdle for building comprehensive datasets. Unsupervised learning techniques do not have this requirement, but in general they are not a good fit for plant pathology recognition, except for generating realistic synthetic images of diseases [24]. In this context, brain–computer interfaces (BCI) emerge as a potential way to accelerate the process by directly tapping into the brain signals of the experts responsible for labeling the data [25].

Artificial intelligence models can achieve some level of generalization and properly recognize patterns under conditions that deviate moderately from those for which they were trained, but they do not come close to the human ability to extrapolate from learned experiences [26]. Although visual assessment of plant diseases can be prone to psychological and cognitive phenomena that may lead to bias and optical illusions [3], these effects become much less prominent with proper training and experience. Using computers to emulate the remarkable human ability to visually recognize patterns has been a long-sought goal. Usually, the first step toward this goal is to record the brain’s response to stimuli relevant to the problem at hand, for example, in the presence or absence of a certain object or event of interest. Non-invasive techniques capable of decoding brain activity include functional magnetic resonance imaging (MRI), magnetoencephalography (MEG), and electroencephalograms (EEG). MRI and MEG require cumbersome, expensive, and non-mobile instrumentation. In contrast, EEG is noninvasive, wearable, and more affordable than other neuroimaging techniques, and is thus a prime choice for any type of practical brain–computer interface (BCI) [27].

The EEG technique has the potential to greatly improve the annotation process. As shown by [25], images can be presented in a very rapid succession (three to ten images per second) and, from the brain signals generated by the user, those images can be tagged with soft labels and then used to train (or retrain) the AI model. As opposed to hard labels, which try to capture the most likely label, soft labels are capable of also capturing the probability associated with the label based on the brain signal response, increasing the generalization capability of the model and reducing overfitting [28,29]. This procedure, called “human-in-the-loop”, not only allows for a large number of images to be labeled in relatively short periods of time, but it can also be combined with the soft labels and active learning techniques to greatly decrease the amount of annotated data needed for training AI models.

EEG techniques can also be used to avoid unreliable results due to fatigue. As demonstrated by studies such as Wang et al. [30], which used EEG signals to detect fatigue in vehicle drivers, and Zhang et al. [31], which employed EEG signals for human emotion recognition, EEG signals can not only be used to annotate the images, but can also give clues about the overall state of the person participating in the process. This enables the inclusion of procedures that interrupt the process and give the subjects some time to rest.

The method proposed in this article improves the results yielded by a deep learning model trained on a limited dataset containing images of healthy and diseased soybean leaves by fine-tuning and retraining the model using the soft labels obtained from the EEG-based system, effectively incorporating the experts’ knowledge into the classification process. This novel approach was carried out in five steps. First, the image-based AI model was trained using a relatively small dataset containing 198 previously labeled samples to serve as baseline. Second, the user profiles for each of the four plant pathology experts involved in the study were established from the respective EEG signals, employing the same 198-sample training dataset. Third, EEG data were collected for each expert on a larger dataset. Fourth, soft labels derived from each expert were assigned to all samples in this larger training dataset. Fifth, the AI model was retrained with this new dataset labeled using only the users’ EEG profiles. Accuracies improved from 96% with the baseline AI model to 99% with the human-in-the-loop and active learning approaches, thus providing strong evidence for the viability of the proposed strategy.

## 2. Material and Methods

### 2.1. Soybean Dataset

The soybean images were captured during the months of January and February 2022 at the experimental fields of Embrapa Soybeans, located in the city of Londrina, Brazil (23.19° W, 51.18° S). The device used for capturing the images was an iPhone 7 mobile phone with resolution of 12 megapixels. Images were labeled by a plant pathologist expert and then used for training and testing the models (Table 1). A total of 1658 unlabeled images were also used in the experiments, as described later in this section.

**Figure 1:**
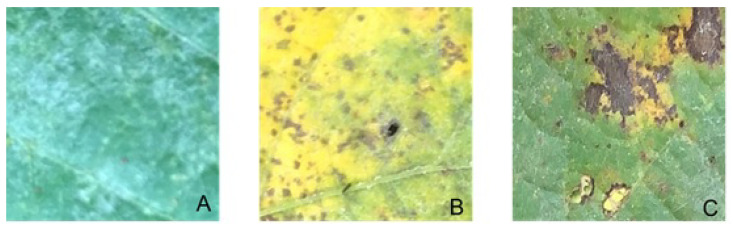
Example of powdery mildew (**A**), rust (**B**), and mix (**C**).

Figure 1 depicts the three types of soybean diseases in the dataset. Powdery mildew symptoms can be identified by various size white powdery spots on the leaf (Figure 1A). Rust symptoms can be identified as yellow/black spots over the leaf surface (Figure 1B). Mixed symptoms can be simply identified as a combination of both rust and powdery mildew symptoms (Figure 1C).

Although two different diseases were considered, the objective of this study was to investigate the potential of models based on EEG signals to accelerate the labeling process as well as to show the advantage of the soft labels in terms of accuracy and robustness. Being the first investigation on the matter, the simpler problem of classifying leaves into “healthy” or “diseased” was tackled, with no attempt at discriminating the diseases at this stage of the research. More complex classification tasks will be investigated in the next steps of the research.

The protocols for image capture were designed to be simple and easy to follow. The only two conditions to be met were that the image should be in focus and the leaf of interest should be the most prominent object in the field of view. More restrictive protocols were not adopted in order to facilitate future practical application of the models. The images cover a wide range of illumination conditions, including different degrees of shadowing, different angles of insolation, and different weather conditions. In addition, a range of different cultivars were imaged, and leaves had different shades of green. Other factors capable of introducing variability, such as different sensors and areas with different weather patterns, will be considered as the research continues.

### 2.2. Initial Training

**Figure 2:**
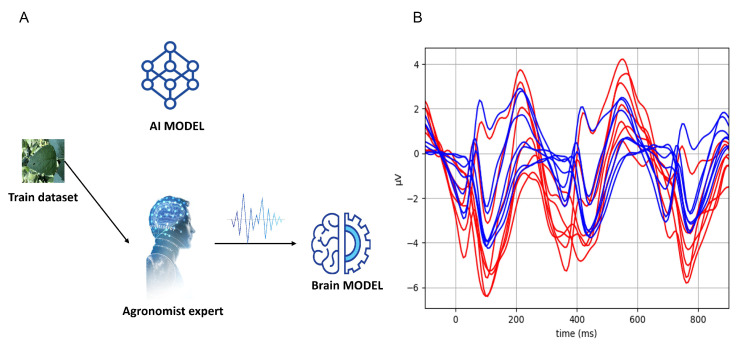
(**A**) Every image from the training set is used to train both the initial AI model and the EEG brain classifier. (**B**) The average plant pathologist brain response to diseased leaves (red) and healthy leaves (blue) across the seven electrodes of the EEG device.

Two separate types of models were initially trained (Figure 2): the first based solely on the images in the training set (baseline AI model), and the second based on the EEG responses of plant pathology experts when presented with images containing healthy and diseased leaves (EEG model).

A convolutional neural network (CNN), pretrained with Imagenet weights (https://www.image-net.org/, accessed on 23 April 2023), was employed for the image-based classification. The adopted architecture used a ResNet50 backbone model and a custom 2D global average pooling. A single neuron with a sigmoid activation function was fine-tuned using the training image dataset described in Table 1. Flips, rotations, shifts, and cropping augmentations were randomly applied using the RandAugment framework [32]. The model was optimized using the ADAM optimizer (initial learning rate of 0.01 and reduce on plateau callback) by minimizing the binary cross-entropy loss function between the model’s prediction and the ground truth. The number of epochs was set to 50 with early stopping callback. The average training time per epoch was 4.87 s.

The data used to train the EEG classifiers were collected using carefully designed steps, as described next. First, the plant pathologists were presented with a rapid stream of images at a 3 Hz rate (i.e., one new image every 333 ms) while wearing an EEG device. They were then instructed to count the occurrences of diseased leaves. Although counting is not strictly necessary, it helps with the subject’s concentration and, as a result, more consistent responses are produced. EEG recordings of four plant pathologists (two from Brazil and two from Israel) were acquired using a DSI-7 EEG headset (https://wearablesensing.com/dsi-7/, accessed on 23 April 2023) at a sampling rate of 300 Hz. The EEG data preprocessing procedure was performed using Python (Scipy v1.4.1, Numpy v1.21.6). First, the recorded EEG was downsampled to 150 Hz in order to increase computational efficiency. Next, a 1–20 Hz bandpass was applied in order to remove slow drifts, prevent aliasing, and clean high-frequency noise. In addition, independent component analysis (ICA) was used to remove artifacts due to eyes blinking [33]. Finally, EEG signals were segmented into 1 s segments, starting 100 ms prior to the image presentation onset and ending 900 ms afterwards. The segmentation process yields a spatiotemporal data matrix with dimensions ($N_{segments}$, $N_{electrodes}$, $N_{timesteps}$) for each expert, where $N_{electrodes}=7$ and $N_{timesteps}=150$. The spatiotemporal matrix is further used to train the EEG based models (one for each expert). Figure 2 (part 2) shows the event related potential (ERP) of one of the plant pathologists.

It is important to notice that the frequency of 3 Hz was chosen after an optimization session conducted with a single plant pathology expert and then adopted for all experts. A possible direction for future research is to investigate if each person produces a different optimal frequency and if it is useful to carry out specific optimization experiments for each new expert. It is also worth noting that the frequency of 3 Hz is considerably above the limits for sustained manual classification of images, even with systems optimized specifically for annotation speed [34].

The system used for collecting the EEG signals adopts some measures to avoid low quality data by monitoring the concentration and attention of the expert using the EEG signals themselves. Basically, the EEG signals generated by the experts are compared with the reference signals determined during the calibration process. If the new EEG signals depart too much from what would be expected for either class (healthy or diseased), which is reflected by low confidence values, the image is reintroduced and new data are collected. If confidence levels remain low, this is a strong indication that the image poses a significant classification challenge even for an expert. Additionally, if the subject shows signs of tiredness, the process is interrupted for at least 5 min for resting. In order to avoid cumulative fatigue, a session never lasted for more than 40 min in total. In addition, no session was carried out right after meals, when alertness tends to drop. A more complete description of the EEG procedure can be found in [27].

Next, EEG classifiers were trained for each plant pathologist expert. The model architecture used in this work was exactly the same described in [35], where all the details about the network’s structure and different elements can be found. In brief, the standard CNN model is decomposed into a series of convolutional and pooling blocks followed by two fully connected layers, creating a more favorable architecture to explore the information contained in the EEG signals. The model was optimized using the ADAM optimizer (variable learning rate, with initial value of 0.03) by minimizing the binary cross entropy loss function between the model’s prediction and the ground truth. The number of epochs was set to 30 with early stopping callback. The average training time per epoch was 2.43 s.

Both the image-based and EEG-based models were implemented using Python and a TensorFlow framework (https://keras.io/, https://www.tensorflow.org/, accessed on 23 April 2023) on a machine with CPU Intel(R) Core(TM) i7-7700 CPU @ 3.60GHz, x86_64, 32GB RAM, GPU GeForce GTX 1080, OS 20.04.4 LTS.

### 2.3. Enhancing the Computer Vision Model with Brain Generated Soft Labels

**Figure 3:**
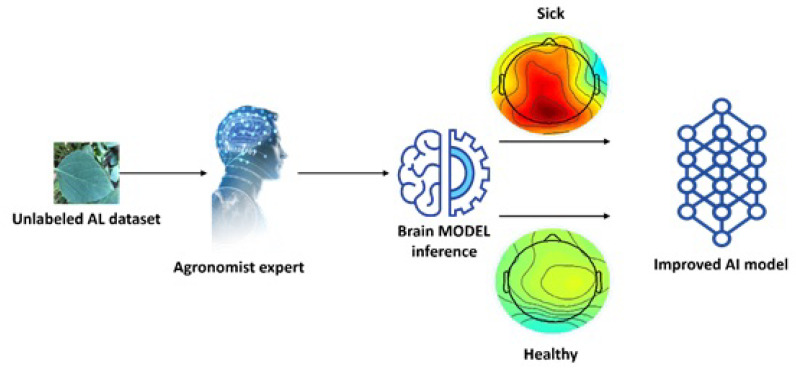
Chosen images from the unlabeled set are rapidly presented to the plant pathologists. The acquired EEG is then used to generate a label and a soft label. The image and associated labels are then used to further train the computer vision model.

As noted in the introduction, the accuracy of the computer vision model can be increased by including images with soft labels generated using the brain signals of the experts [36]. Traditionally, the newly labeled images are used to retrain the computer vision model. However, in order to better utilize the plant pathologist knowledge, EEG signals were combined with the computer vision model using a procedure called human-in-the-loop (HITL). As illustrated in Figure 3, a subset of the unlabeled set selected using active learning is shown to the plant pathologist [25], and the generated soft labels are used to retrain the model. Soft labels (a score between 0 and 1) can be interpreted as the plant pathologist’s confidence level regarding the disease detection. The adoption of soft labels makes it possible to give more weight to images with high confidence scores while also exploring the information yielded by images whose scores are more uncertain (close to the decision threshold). As shown in Peterson et al. [28], the uncertainty involved in the classification performed by humans can be very valuable to increase the robustness of the model.

**Figure 4:**
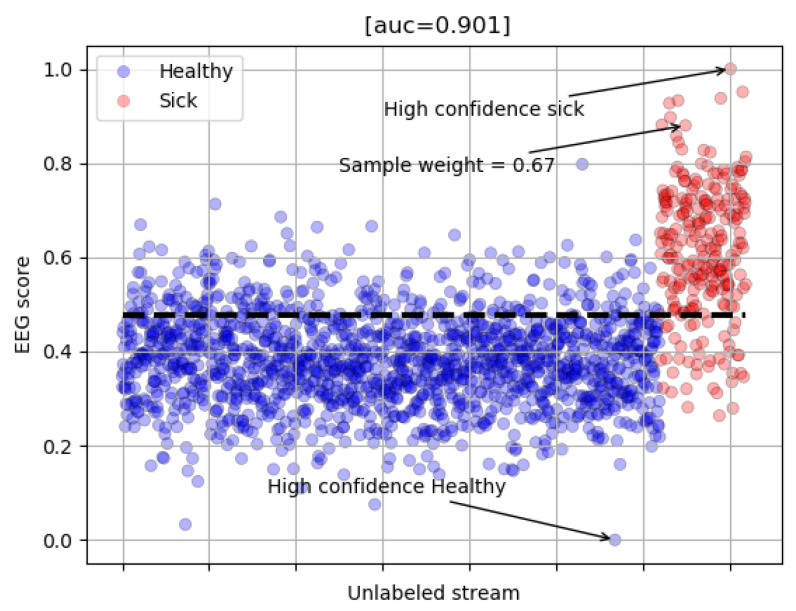
Soft scores generated for one of the plant pathology experts.

Figure 4 shows the soft label scores (s) generated using the EEG signals of one of the experts. Scores closer to the central portion of the plot (dashed line, t) indicate low confidence, and the corresponding images are assigned lower sample weights (w) when retraining the model. On the other hand, values closer to the extremities of the plot indicate high confidence, so the corresponding images are mapped into higher training weights. The function that maps the EEG score to the sample weight is given by the following pseudocode:

(1) $$w=\left\{\begin{array}{cc} \frac{t-s}{t} & \mathrm{if}\;s\geq t\\ \frac{s-t}{1-t} & \mathrm{otherwise} \end{array}\right.$$

### 2.4. Experiments

Before the experiments, all images containing diseased leaves were grouped into a single class, because at this stage of the research the objective was to validate the new methodology, and not to stress the system’s capabilities with the more challenging task of discriminating between diseases. In order to determine the effectiveness of the proposed method, five experiments were carried out:

(1) Training and evaluation of the baseline AI model. Three different CNN architectures, ResNet-50, MobileNet, and EfficientNet, were tested. As noted in Section 2.1, only 198 images were used for training. This setup aimed at emulating the very common situation in which the number of labeled images is usually small and limited, whereas the number of unlabeled images is large and unconstrained.
(2) Training and evaluation of the EEG models. Five models were created for each expert (5-fold cross-validation) in order to produce more realistic results and get a sense of reproducibility.
(3) Training and evaluation of the improved AI model by incorporating the models associated with each expert separately. Again, 5-fold cross-validation was adopted.
(4) Training and evaluation of the improved AI model by incorporating the models associated with each expert and then averaging the results in order to produce a single combined classification for each test sample.
(5) Experiment 4 was repeated using active learning and brain-in-the-loop concepts. The objective was to determine if this approach would reduce the number of samples needed to achieve the accuracy yielded by the “offline” approach.

The performance of the described setups were measured in terms of an ROC-AUC metric, which effectively combines false negative and false positive ratios into a single value. AUC is a commonly used statistic used for model comparison [37]. AUC values can range from 0 to 1; however, an AUC of 0.5 indicates the corresponding classification model is basically worthless, as its predictive ability is no better than random guessing. An AUC value of 1 indicates perfect classification. Values between 0 and 0.5 imply worse classification than random guess and are usually ignored.

Visual inspection of the EEG curves was also carried out in order to determine if there was indeed a clear difference between healthy and diseased samples.

## 3. Results and Discussion

### 3.1. AI Model

The ResNet-50 model was chosen for its combination of a compact architecture in terms of number of trainable parameters with good generalization capabilities when dealing with small datasets. Other architectures were considered (EfficientNet-b0, MobileNet), but after short empiric examination, no significant differences were observed. Several combinations of hyperparameters were tested, including different dimensions for the input images, with the size of 448 × 448 pixels being finally adopted instead of the standard 224 × 224 pixels, as it yields better results without excessively increasing training times (Table 2).

The AUC obtained using the baseline AI model was 0.968. Although this already indicates a high accuracy level, it is worth noting that most errors are due to images for which symptoms are slight and ambiguous. This is important because an automatic method for disease detection and recognition will be more useful exactly at the earlier stages of development, when symptoms are still mild and severe damage can still be prevented. In addition, even when symptoms are well set, they may present ambiguous characteristics that might not be easily recognized by someone without extensive training. Thus, even small improvements in the accuracy can have a great impact on the usefulness of the method.

### 3.2. EEG Models

Table 3 shows the results obtained using solely the EEG models generated for each expert. No expert had any noticeable eyesight problems, and all showed similar abilities in recognizing fainter symptoms in the images. Thus, the significant differences observed between experts are likely due to individual psychological and cognitive characteristics and the level of comfort using the EEG equipment. However, a definitive answer for this phenomenon can only be achieved through additional experiments. It is worth noting that the standard deviation of the different classifiers is relatively low, suggesting that EEG classifiers are stable and reproducible.

### 3.3. Improved AI

Despite the EEG classifiers heterogeneity between experts, the inclusion of the individual EEG models into the AI model training tended to yield higher accuracies (Table 4). This fact supports the claim that incorporating human uncertainty into the model usually increases its robustness [28].

**Figure 5:**
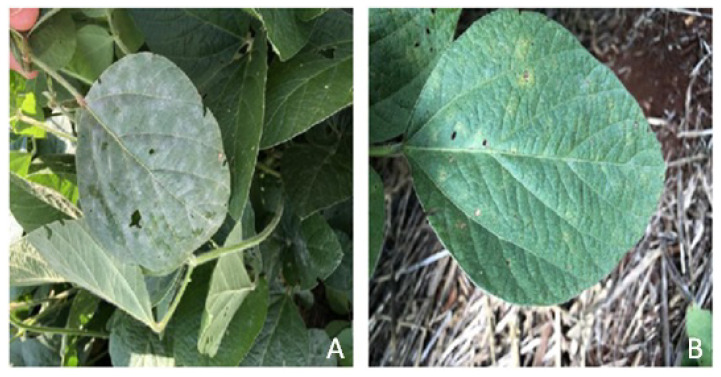
Examples of misclassifications that were corrected by the incorporation of expert knowledge. Because of the limited variability found in the training set, uncommon conditions such as the excessive presence of leaves (**A**) and stubble (**B**) caused the baseline model to fail.

Figure 5 shows two examples of images that were originally misclassified by the baseline model but were correctly classified by the improved AI model.

### 3.4. Panel of Experts

**Figure 6:**
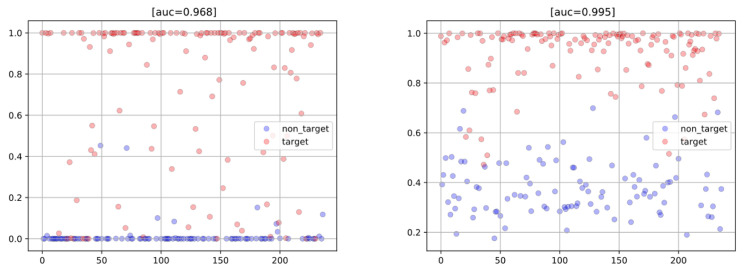
Scatterplots obtained using the baseline model (**left**) and the improved AI model (**right**). Red and blue circles represent diseased and healthy leaves, respectively. The vertical axis shows the score yielded by the model for each sample.

The AUC of the improved AI model increased to 0.995 when the EEG models of all four experts were included. Figure 6 shows a graphical comparison between the results obtained by the baseline and improved AI models. In these scatterplots, the closer the score is to 0, the larger the confidence that the sample is healthy. Conversely, the closer the score is to 1, the larger the confidence that the sample is diseased. The threshold between healthy and diseased was set at 0.5. As can be seen, not only is the number of misclassifications smaller for the improved model, but also the distribution of the scores is less dichotomic and more scattered. This more closely resembles the behavior of human experts and, more importantly, indicates an increased robustness to new data, as “over-confident” models tend to perform poorly in this regard [38,39,40].

### 3.5. Brain-in-the-Loop

**Figure 7:**
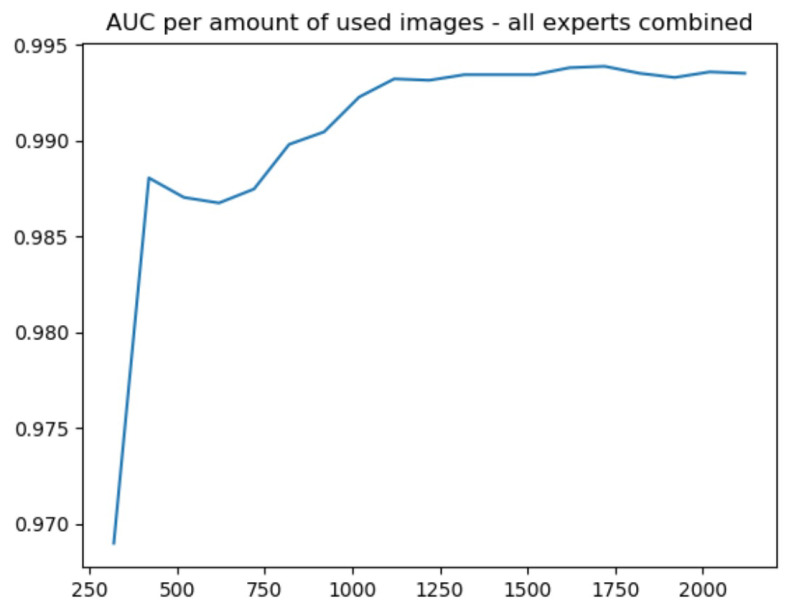
AUC versus number of brain-in-the-loop number of samples.

Finally, the active learning plus brain-in-the-loop approach was applied. As indicated in Figure 7, the number of samples needed to reach AUC = 0.995 fell from 2000 in the original experiment to 1100.

### 3.6. Additional Remarks

The experiments carried out in this study tackled a binary classification problem using a relatively limited image dataset. Although the models generated would not be useful for practical use, the conclusions drawn from the results yielded by them provided compelling evidence that coding expert knowledge in the form of EEG-based models can at least partially solve data-related problems that still hamper the practical application of many AI-based technologies in agriculture [4]. In particular, the labeling process can become more agile, and the incorporation of the human uncertainty into the models has the potential to make those more robust to conditions that are underrepresented in the data used for training. Notwithstanding the success achieved so far, there are some crucial questions that require further investigation.

Question 1: How well will the EEG-based approach adapt to a multi-class classification problem? Although a simple warning that something is wrong with a given plant without specifying the problem is already useful, the ability to discriminate the disease can be very valuable and enable a number of helpful technologies. As powerful as the techniques applied here are, it is unlikely that they can be applied to a large number of diseases due to separability issues. Nevertheless, in principle it seems possible to tackle at least the most impactful diseases. Future research will try to determine how separable are the signals generated by different diseases, as well as the maximum number of diseases that can be reliably considered.

Question 2: How robust is the improved AI model? The experiments carried out so far provided strong evidence that the inclusion of expert knowledge and associated uncertainties indeed improves the robustness of the model to new data. However, the dataset used in the tests has a relatively limited variability, so the robustness of the model was not severely stressed. Future experiments will explore this issue by testing the model with completely independent data. It is worth pointing out that with the active learning approach, samples for which the model tends to fail can be used for retraining, thus continuously improving its accuracy.

Question 3: What is the optimal number of experts? As discussed earlier, the soft labels of an individual expert can capture the probability associated with an image, thus, soft labels from a panel of experts can capture the distribution associated with an image. In the experiments carried out in this study, the inclusion of additional experts using their soft labels’ arithmetic mean was always advantageous. However, it is not clear if this is the case indefinitely. Although further investigation is required, the soft label distribution can be aggregated in more sophisticated ways, for example, an average weighted by the expert’s concentration levels obtained from the EEG.

## 4. Conclusions

This article presented a study on the use of brain signals from plant pathology experts to improve the results yielded by image-based AI models for plant disease recognition. Experiments with images of soybean leaves affected by powdery mildew and rust have shown that incorporating soft labels representing the knowledge encoded in the expert’s brains while monitoring the respective attention levels not only improves the classification accuracy, but also boosts the robustness of the AI model to new data. In addition, the brain-in-the-loop procedure led to a reduction in the amount of labeled data required to achieve good results, saving precious expert time. Future research will focus on the discrimination of different diseases and on answering questions regarding the robustness of the model and the ideal number of experts.

## Figures and Tables

**Table 1:** Soybean image datasets used in the experiments.

Disorder	# Training Samples	# Test Samples
Healthy	148	101
Rust	34	93
Powdery mildew	12	33
Mix	4	10

**Table 2:** Effect of the image size on the AUC (validation set) and the average epoch time.

Image Size	112 × 112	224 × 224	448 × 448
Average epoch time	2.1 s	2.14 s	4.87 s
AUC	0.929	0.941	0.948

**Table 3:** AUC values obtained for each expert.

Expert	Fold 1	Fold 2	Fold 3	Fold 4	Fold 5	Mean	Std
1	0.875	0.855	0.861	0.866	0.877	0.867	0.009
2	0.828	0.821	0.823	0.830	0.833	0.827	0.005
3	0.701	0.739	0.731	0.709	0.710	0.718	0.016
4	0.650	0.674	0.689	0.628	0.637	0.656	0.025

**Table 4:** AUC values obtained by the AI models enhanced by EEG classifiers.

Expert	Fold 1	Fold 2	Fold 3	Fold 4	Fold 5	Mean	Std
1	0.988	0.986	0.979	0.989	0.988	0.986	0.004
2	0.983	0.986	0.972	0.987	0.983	0.982	0.006
3	0.957	0.982	0.958	0.970	0.935	0.960	0.017
4	0.974	0.978	0.993	0.932	0.972	0.970	0.023

## Data Availability

Data available on request due to restrictions. This is due to the intention of using the method described in this article as basis of a future commercial product.
